# Brain nonapeptide levels are related to social status and affiliative behaviour in a cooperatively breeding cichlid fish

**DOI:** 10.1098/rsos.140072

**Published:** 2015-02-04

**Authors:** Adam R. Reddon, Constance M. O'Connor, Susan E. Marsh-Rollo, Sigal Balshine, Magdalena Gozdowska, Ewa Kulczykowska

**Affiliations:** 1 Aquatic Behavioural Ecology Laboratory, Department of Psychology, Neuroscience, and Behaviour, McMaster University, 1280 Main Street West, Hamilton, Ontario, Canada L8S 4K1; 2 Genetics and Marine Biotechnology, Institute of Oceanology of Polish Academy of Sciences, Powstanców Warszawy 55 Street, 81-712 Sopot, Poland

**Keywords:** isotocin, arginine vasotocin, oxytocin, vasopressin, dominance, *Neolamprologus pulcher*

## Abstract

The mammalian nonapeptide hormones, vasopressin and oxytocin, are known to be potent regulators of social behaviour. Teleost fishes possess vasopressin and oxytocin homologues known as arginine vasotocin (AVT) and isotocin (IT), respectively. The role of these homologous nonapeptides in mediating social behaviour in fishes has received far less attention. The extraordinarily large number of teleost fish species and the impressive diversity of their social systems provide us with a rich test bed for investigating the role of nonapeptides in regulating social behaviour. Existing studies, mostly focused on AVT, have revealed relationships between the nonapeptides, and both social behaviour and dominance status in fishes. To date, much of the work on endogenous nonapeptides in fish brains has measured genomic or neuroanatomical proxies of nonapeptide production rather than the levels of these molecules in the brain. In this study, we measure biologically available AVT and IT levels in the brains of *Neolamprologus pulcher*, a cooperatively breeding cichlid fish, using high performance liquid chromatography with fluorescence detection. We found that brain AVT levels were higher in the subordinate than in dominant animals, and levels of IT correlated negatively with the expression of affiliative behaviour. We contrast these results with previous studies, and we discuss the role the nonapeptide hormones may play in the regulation of social behaviour in this highly social animal.

## 2. Introduction

Understanding the mechanistic underpinnings of social behaviour represents a major research goal in behavioural biology [1–3]. The nonapeptide hormones, oxytocin and vasopressin, have attracted a great deal of research attention, and there is strong evidence that these neurohormones are key proximate regulators of mammalian social behaviour [4–9]. These neuropeptides are evolutionarily ancient molecules that trace back to the original nonapeptide, arginine vasotocin (AVT), which is found in extant non-mammalian vertebrates [10]. Oxytocin, which originally arose from a duplication of the AVT gene early in the vertebrate lineage, also has non-mammalian homologues in all extant vertebrates, including isotocin (IT) in teleost fishes and mesotocin in birds, amphibians and non-avian reptiles [10,11]. Much less research attention has been dedicated to the role that these homologous molecules play in the regulation of social behaviour in non-mammalian vertebrates, though in recent years, this has begun to change, notably with work that has been done linking AVT and mesotocin to social behaviour in birds [12–15].

Teleost fishes represent by far the most diverse group of living vertebrates [16] and exhibit a similarly diverse range of social systems [17]. Teleost fishes, therefore, present an excellent opportunity to investigate the role of the nonapeptide hormones in regulating social behaviours [18]. Furthermore, extant teleosts may provide a window into the ancestral role of nonapeptide circuits in regulating social behaviour, and offer insights into the degree to which nonapeptide hormone function has been conserved through vertebrate evolution [10,19]. Despite the remarkable diversity of the teleost fishes, relatively little work has been done to investigate how these nonapeptide hormones influence the social behaviour of fishes [18,19].

The relatively small number of experiments that have manipulated AVT and IT levels in fishes have provided some clues as to the function of these neuropeptides in regulating social behaviour. For example, nonapeptides appear to regulate social approach behaviour in fishes. Goldfish (*Carassius auratus*) that received an intracerebroventricular infusion of AVT spent less time associating with a conspecific, whereas fish that received an administration of an AVT antagonist spent more time near a conspecific [20]. An intracerebroventricular infusion of IT also increased social approach in the same species [20], suggesting that both AVT and IT play a role in the regulation of social approach in goldfish. Consistent with that conclusion, both AVT and IT affect social approach when given peripherally to zebrafish (*Danio rerio*), with the direction of those effects being dose-dependent [21]. The administration of exogenous AVT increases aggression in bluehead wrasse (*Thalassoma bifasciatum* [22]), brown ghost knifefish (*Apteronotus leptorhynchus* [23]) and beaugregory damselfish (*Stegastes leucostictus* [24]), but decreases aggression in Amargosa River pupfish (*Cyprinodon nevadensis* amargosae [25]) and zebrafish (*Danio rerio* [26]). Administrations of IT, by contrast, seem to have little effect on the absolute level of aggressive behaviour in fishes [24,27]. Nonapeptide administrations also have effects on pair bonding and parental care behaviours in the monogamous convict cichlid (*Amatitlania nigrofasciata*). A non-specific AVT/IT antagonist delivered peripherally delayed, but did not prevent, pair bonding in convicts [28], whereas a selective IT antagonist interfered with parental care [29].

Most of the previous studies in fishes have used indirect measures of nonapeptide concentrations based on neuroanatomy or mRNA expression to examine the relationship between endogenous nonapeptide levels in the brain and social behaviour. These studies have used the techniques such as immunohistochemistry, *in situ* hybridization or qPCR. These studies have provided some insights into the relationship between endogenous nonapeptides in the brain and social behaviour in fishes, and have shown that individual neuronal phenotypes are predictive of differences in behaviour and dominance status. For example, in the plainfin midshipman, *Porichthys notatus*, dominant territorial males have larger AVT-immunoreactive cells in their preoptic area compared with females or sneaker males [30]. In the beaugregory damselfish, AVT fibre density in the parvocellular region of the preoptic area is inversely related to aggression [31]. In the polygynous African cichlid, *Astatotilapia burtoni*, dominant males show greater AVT mRNA expression in the gigantocellular region of their preoptic area, whereas subordinate males have greater expression in the parvocellular region of the preoptic area [32]. Interestingly, AVT expression in dominant males correlates positively with aggressive behaviour, whereas AVT expression in the subordinate males correlates positively with submissive behaviour [32].

Recently, high performance liquid chromatography with florescence detection (HPLC-FL) has been used to measure the levels of nonapeptide hormones directly within fish brains [33]. Such measurement of biologically available molecules may be more straightforwardly related to the current neuromodulatory actions of nonapeptides in the animal’s brain compared with indirect proxies of nonapeptide production. Using this technique, fishes have been shown to differ in the amount of AVT and IT in their brains depending on dominance and reproductive status. Subordinate *Oreochromis mossambicus* had higher levels of AVT in their pituitary and higher levels of IT in their hindbrain than did dominant fish [34]. Conversely, both AVT and IT levels were higher in whole brain preparations of dominant three-spined stickleback males, *Gasterosteus aculeatus*, relative to socially subordinate animals [35]. Moreover, female three-spined sticklebacks housed alone have higher AVT and lower IT concentrations in their brains relative to females housed in groups, whereas males housed alone have increased AVT levels and show no differences in IT concentrations relative to males housed in groups [36]. Biologically available nonapeptide levels in the brain also differ depending on social housing density in the round goby (*Neogobius melanostomus*), where non-aggressive males held at high densities show higher whole brain AVT and lower whole brain IT levels than territorial males housed at lower density [37].

In order to further explore the relationship between nonapeptide levels in fish brains and social behaviour, we made use of an emerging model system in the integrative biology of social behaviour, the cooperatively breeding cichlid fish, *Neolamprologus pulcher*. *Neolamprologus pulcher* are a substrate spawning cichlid fish endemic to the rocky littoral zone in Lake Tanganyika, East Africa [38]. *Neolamprologus pulcher* exhibit a complex social system characterized by frequent social interactions and a suite of specialized social behaviours and signals [39–43]. *Neolamprologus pulcher* live and breed within social groups comprising a single dominant breeding pair and 1–20 non-breeding adult subordinates of both sexes [42,44]. Subordinate *N. pulcher* assist the reproductive efforts of the dominant pair by clearing the territory of sand and debris, defending against predators, conspecific and heterospecific space competitors, and participating in care of the young (for review, see [43]). The exceptional social nature of this fish combined with its tractability for controlled laboratory experiments makes *N. pulcher* an attractive study system for unravelling the role the nonapeptide hormones play in regulating social behaviour [27]. In particular, *N. pulcher* are small-bodied (maximum size is approx. 80 mm standard length), fast-growing and adapt well to life in aquaria [40,45] where they form naturalistic social groups and perform their full suite of social behaviours [46].

Previous studies examining the functions of the nonapeptides in *N. pulcher* suggest that these hormones play an important role in regulating social behaviour in this species. In a microarray study, dominant *N. pulcher* showed higher expression of the AVT gene than did subordinates, suggesting that AVT plays a role in determining social status in this species [47]. Reddon *et al.* [27] found that *N. pulcher* treated with an intraperitoneal injection of IT showed more submissive displays within their social groups. Submissive displays are an important social signal in this species, which may serve to appease dominant group members [48] and facilitate conflict resolution while allowing both parties to remain in the same spatial location [49], suggesting a possible role for IT in the regulation of the dominance hierarchy. Reddon *et al.* [50] found that exogenous IT reduced the tendency for *N. pulcher* to approach and affiliate with conspecifics, in contrast to previous work in goldfish [20].

In this study, we investigated the relationship between nonapeptide hormones, dominance status and social behaviour in *N. pulcher*. Following behavioural observations, we measured the concentrations of biologically available nonapeptides, AVT and IT, in the brains of dominant and subordinate *N. pulcher* of both sexes using HPLC-FL. The results of this study complement previous work analysing nonapeptide gene expression and manipulating nonapeptides in *N. pulcher* [27,47,50] and together offer a more complete picture of the role of the nonapeptide hormones in regulating social behaviour in this highly social fish.

## 3. Material and methods

### 3.1 Study animals

All of the fish that we used in this study were adults from a breeding colony of *N. pulcher* held at McMaster University, Hamilton, Ontario, Canada. The fish were descendants of breeding pairs caught in Lake Tanganyika, Zambia in 2002 and 2008 and had been housed in the laboratory for several generations prior to this study. The fish lived in social groups consisting of a male and female dominant breeding pair with two subordinate helpers. These social groups had been together for at least one month prior to the onset of this study, and all groups had successfully reproduced at least once before being included in this study. Therefore, these represent stable social groups. Each social group was housed in a 189 l freshwater tank outfitted with a heater, thermometer, two foam filters, approximately 2 cm of coral sand substrate, and two inverted flowerpot halves for use as shelters and nest sites. The light : dark cycle was kept constant at 13 : 11 h, and water temperature was maintained at 26±2°C. We fed the fish ad libitum 6 days per week with Hagen Nutrafin Basix flake cichlid food.

### 3.2 Study protocol

At the beginning of the study, we identified and captured the dominant breeding pair and the two subordinate helpers from each social group (*n*=10). We sexed the fish by examination of their external genitalia, measured (standard length and mass; table 1), and uniquely fin-clipped them before returning them to their home tanks. These fin clips are temporary, and the removed fin tissue regrows within a fortnight. Fin clipping does not adversely affect behaviour [51], and fish quickly recover from this procedure. We carried out two detailed behavioural observations per group on consecutive days one week after measuring and marking the fish. All 10 groups were observed in random order on the same 2 days. Two trained observers observed each group once on each day for 10 min between 10.00 and 13.00. We recorded all social behaviours (aggressive, submissive and affiliative) for all individuals in each group. The behaviours recorded are described in detail in an ethogram for this species (table 2; adapted from [49,52]). Briefly, the behaviours we saw in our focal groups were aggression, submission (submissive head-up posture, submissive displays, hook displays) and affiliation (follows, parallel swims, soft touches). We further subdivided the aggressive behaviour into overt aggression (chases, rams, bites) and restrained aggression (head-down postures, frontal displays).

**Table 1.** Sample sizes, masses and social behaviour for all *Neolamprologus pulcher* individuals included in the current study. (All values are presented as the median and the interquartile range (i.e. the 50th percentile (25th–75th percentile)).)

status	sex	sample size	mass (g)	SL (mm)	brain mass (mg)	overt aggression (acts ⋅ 10 min^−1^)	restrained aggression (acts ⋅ 10 min^−1^)	submission (acts ⋅ 10 min^−1^)	affiliation (acts ⋅ 10 min^−1^)
breeder	male	9	10.0 (9.6–11.2)	71.6 (69.6–72.4)	40.9 (37.4–41.2)	0.5 (0.5–1.0)	9.5 (6.0–10.0)	0.0 (0.0–0.0)	1.0 (0.0–1.5)
breeder	female	9	7.9 (7.4–8.9)	64.9 (62.4–68.8)	38.2 (34.7–42.0)	0.0 (0.0–0.0)	3.5 (2.5–8.0)	2.5 (2.0–3.5)	3.0 (1.5–5.5)
subordinate	male	9	3.4 (1.8–3.6)	47.6 (40.5–51.1)	2.63 (2.17–2.97)	0.0 (0.0–0.0)	0.0 (0.0–1.5)	4.5 (4.0–7.5)	1.5 (0.5–5.5)
subordinate	female	11	6.1 (4.8–7.0)	59.0 (55.6–61.0)	3.18 (2.95–3.49)	0.0 (0.0–0.0)	1.0 (0.5–3.5)	4.0 (2.3–8.0)	2.5 (0.8–3.5)

**Table 2.** The behaviours produced by *Neolamprologus pulcher* in the current study. (This ethogram is based on extensive laboratory and field observations, and adapted from Sopinka *et al.* [52] and Hick *et al.* [49].)

context	behaviour	description
overt aggression	chase	focal fish quickly darts towards another fish
	ram	focal fish makes contact with another fish using the head or mouth region, but no obvious bite is taken and jaws remain closed
	bite	focal fish bites another fish
restrained aggression	aggressive posture	focal fish lowers its head and raises its tail in front of its opponent. Unpaired fins are held erect
	frontal displays	also called a puffed throat or an opercular flare. Focal fish extends out its opercula and lower jaw. Often associated with a posture where the head is pointed downwards
submission	submissive posture	the head of the focal fish is directed upwards, sometimes the body is held entirely vertical, and the tail is downwards
	submissive display	focal fish is positioned with a submissive posture accompanied by a quivering tail. Sometimes the entire body quivers
	hook display	also known as a J-display. Focal fish swims towards another fish, and then turns sharply away at the last moment and quivers
affiliation	following	focal fish follows another closely
	parallel swim	both fish swim upwards together in a parallel fashion in close proximity
	soft touch	focal fish nips or softly contacts another individual

Immediately following the final behavioural observation, we quickly captured the focal fish, stunned them in an ice-water bath for 5–10 s and then killed them by spinal severance. We extracted the whole brain from each fish within 2 min post-mortem. We weighed (table 1) and then immediately froze each brain on dry ice, and then stored them at −80°C until the analyses of AVT and IT concentrations were conducted. We confirmed the sex of each fish anatomically during dissection.

### 3.3 Brain isotocin and arginine vasotocin assays

We determined the AVT and IT content in the brains of *N. pulcher* using HPLC-FL preceded by solid-phase extraction (SPE). The frozen brains were thawed and weighed before being sonicated in 1 ml Milli-Q water (MicrosonXL, Misonix, Farmingdale, NY). We added glacial acetic acid (3 μl) to the homogenates, and then placed the samples in a boiling water bath for 3.5 min. The extracts were cooled on ice, and then centrifuged at 8000*g* for 20 min at 4°C. We decanted the supernatants and loaded onto preconditioned (1 ml MeOH, 1 ml distilled water) SPE columns (30 mg ml^−1^, STRATA-X, Phenomenex, Torrance, CA). We passed water (600 μl) and then 0.1% trifluoroacetic acid (TFA) in 5% acetonitryl (600 μl) through the columns to wash away impurities, and then eluted the peptides by 2×600 μl of 80% acetonitrile. The eluate was evaporated using Turbo Vap LV Evaporator (Caliper Life Science, Hopkinton, MA). We then froze the samples and stored them at −80°C prior to HPLC analysis.

Before quantitative analysis, we re-dissolved the samples in 50 μl of 0.1% TFA in 30% acetonitrile and divided them into two aliquots. The derivatization of AVT and IT in each of the 25 μl samples was preformed using 3 μl of 4-fluoro-7-nitro-2,1,3-benzoxadiazole (NBD-F) solution (30 mg NBD-F in 1 ml of acetonitrile) in 25 μl phosphoric buffer (0.2 M, pH 9.0). We heated the solution at 60°C for 3 min in a dry heating block and then cooled it down on ice before adding 5 μl of 1 M HCl. We measured the derivatized samples with Agilent 1200 Series Quaternary HPLC System (Agilent Technologies, Santa Clara, CA). Chromatographic separation was achieved on a ZORBAX Eclipse XDB-C18 column (Agilent Technologies, 150×4.6 mm I.D., 5 μm particle size). A gradient elution system was applied for separation of derivatized peptides. The mobile phase consisted of solvent A (0.1% TFA in H_2_O) and solvent B (0.1% TFA in acetonitrile : H_2_O [3 : 1]). A linear gradient was 40–65% of eluent B in 20 min. We set the flow rate at 1 ml min^−1^ and the column temperature at 20°C. The injection volume was 58 μl. The fluorescence detection was carried out at 530 nm with excitation at 470 nm.

Our procedure allowed us to determine the concentration of free AVT and IT after their dissociation from non-covalent complexes. This is important, because only this nonapeptide fraction binds to nonapeptide receptors allowing them to act as neurotransmitters and/or neuromodulators in the brain. This analytical procedure, which permits the measurement of biologically active nonapeptides at their site of action, has been used successfully, with slight modifications, in several fish species [33–37,53].

### 3.4 Statistical analysis

We summed all overt aggressive behaviours, restrained aggressive displays, submissive displays, and affiliative behaviours produced by each individual across both observation periods and then calculated the rate of each class of behaviour produced per minute for each individual (table 1). We then ran linear models to explore the influence of each measured class of social behaviour produced per individual on the levels of neuropeptides. We also examined the effect of sex (male or female) and dominance status (breeder or subordinate), on brain levels of AVT and IT. We initially included all two-way interactions in the models, but none were significant (*α*=0.05) and these were dropped from the final models. Social group identity was included as a random effect in all models. Concentrations of AVT and IT were log-transformed to meet the assumptions of the parametric tests. This study is correlational, and we cannot speak to the causal direction for the relationships we report. We treated our nonapeptide measures as the response variable in the analyses and figures, because we collected these measurements after the behavioural and demographic data. All analyses were performed using R v. 2.15.1.

## 4. Results

**Figure 1.**
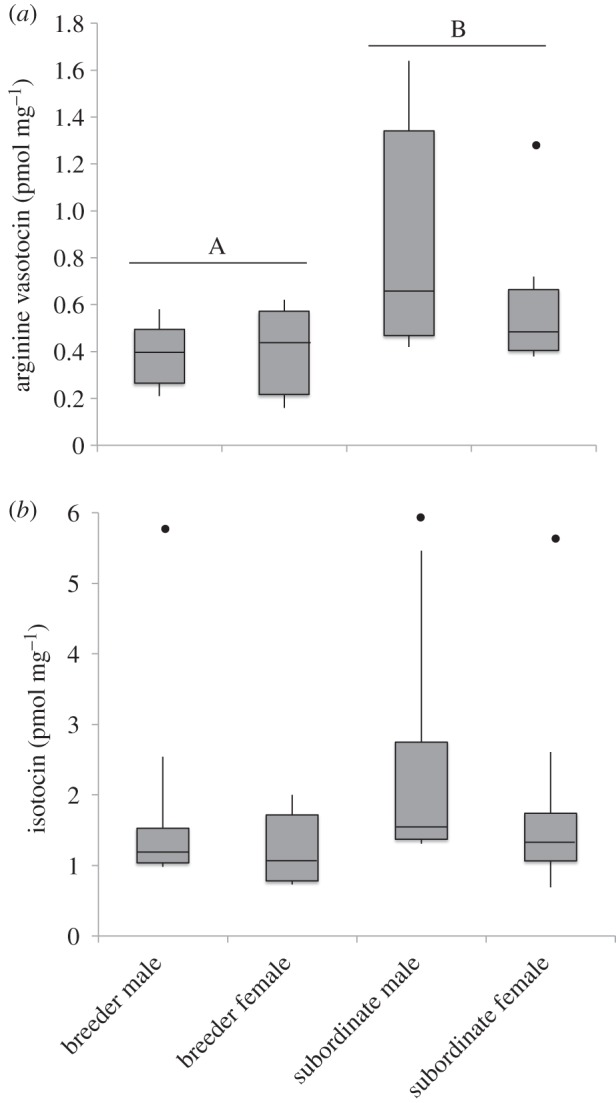
The relationship between (*a*) brain arginine vasotocin (AVT) and (*b*) brain isotocin (IT) concentration, and sex and dominance status in *Neolamprologus pulcher*. Subordinate *N. pulcher* have higher brain AVT concentrations. Boxes represent the interquartile range (i.e. the 25th–75th percentile) of the data, with the median denoted by the band inside the box. Whiskers represent the 10–90th percentile of the data. Outliers are indicated. For sample sizes, see table 1. For full statistical details, see table 3.

Brain AVT concentrations were consistently higher in the subordinate than in the dominant *N. pulcher* (table 3 and figure 1*a*). There was a trend towards higher brain IT concentrations in the subordinates relative to dominant individuals and in males relative to females, but these trends did not reach significance (table 3 and figure 1*b*).

**Table 3.** Results of linear models exploring the influence of sex, status and behaviour on brain arginine vasotocin (AVT) and isotocin (IT) concentrations in *Neolamprologus pulcher*. (Two-way interaction terms were included in the original models, but none were significant (*α*=0.05), and so these were dropped from the final models presented here. Values in italics indicate non-significant trends (0.1<*p*>0.05). Values in bold-italics indicate model terms that significantly contribute to significant models (*p*<0.05).)

nonapeptide	model term	estimate	standard error	*t*-ratio	*p*-value
AVT	overt aggression	0.19	0.17	1.13	0.27
	sex	0.20	0.18	1.10	0.28
	***status***	***0***.***59***	***0***.***19***	***3***.***11***	***0***.***004***
	restrained aggression	0.002	0.03	0.08	0.93
	sex	0.25	0.18	1.38	1.18
	***status***	***0***.***05***	***0***.***23***	***2***.***19***	***0***.***03***
	*submission*	−*0*.*03*	*0*.*02*	−*1*.*93*	*0*.*06*
	sex	0.18	0.17	1.07	0.29
	***status***	***0***.***66***	***0***.***18***	***3***.***62***	***0***.***001***
	affiliation	−0.04	0.04	−0.91	0.37
	sex	0.24	0.18	1.35	0.19
	***status***	***0***.***52***	***0***.***18***	***2***.***93***	***0***.***007***
IT	overt aggression	d0.03	0.18	−0.21	0.83
	*sex*	*0*.*33*	*0*.*19*	*1*.*71*	*0*.*09*
	status	0.25	0.20	1.21	0.24
	*restrained aggression*	−*0*.*05*	*0*.*03*	−*1*.*89*	*0*.*07*
	*sex*	*0*.*37*	*0*.*18*	*2*.*09*	*0*.*05*
	status	−0.02	0.23	−0.10	0.92
	submission	−0.02	0.02	−1.01	0.32
	sex	−0.29	0.18	1.58	0.13
	*status*	*0*.*36*	*0*.*20*	*1*.*76*	*0*.*09*
	***affiliation***	−***0***.***09***	***0***.***04***	−***2***.***41***	***0***.***02***
	sex	0.28	0.17	1.65	0.11
	*status*	*0*.*32*	*0*.*17*	*1*.*87*	*0*.*07*

**Figure 2.**
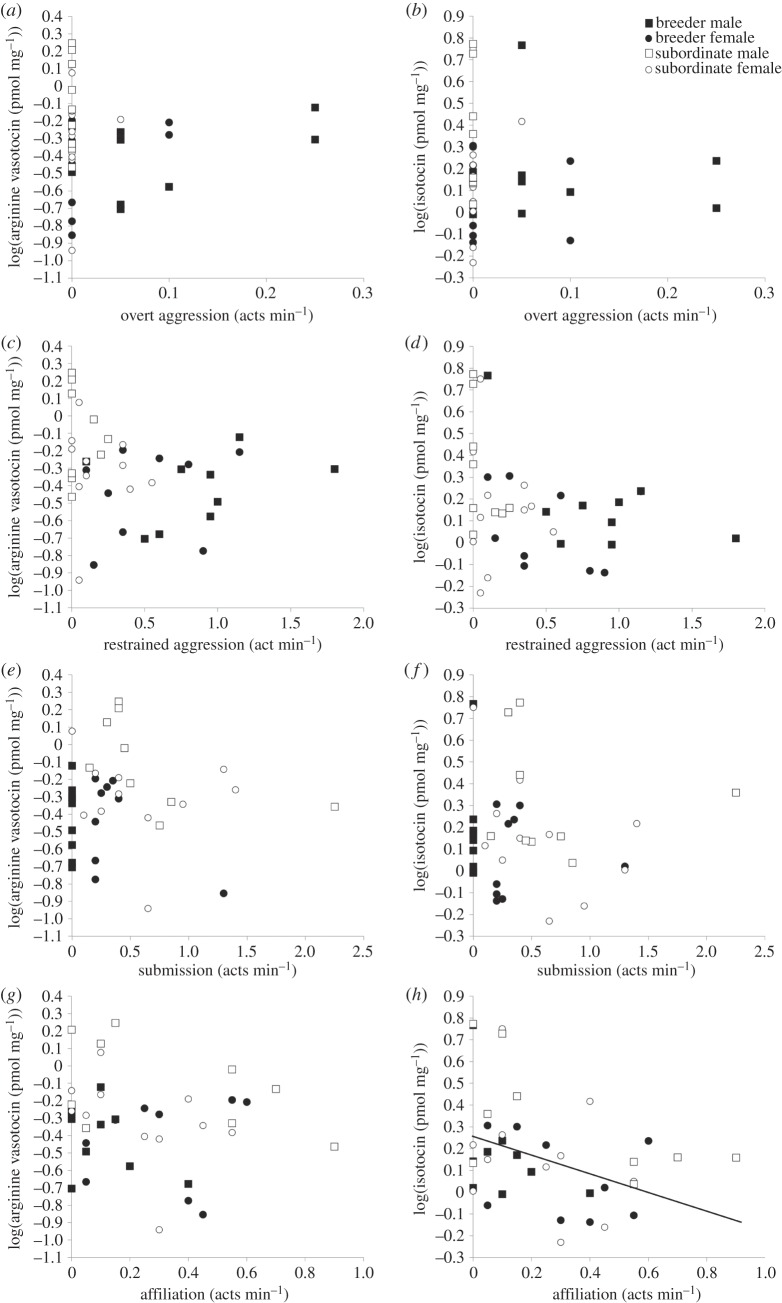
The relationship between brain arginine vasotocin (AVT) and brain isotocin (IT) concentration and overtly aggressive (*a*,*b*), restrained aggressive (*c*,*d*), submissive (*e*,*f*), and affiliative (*g*,*h*) behaviours in *Neolamprologus pulcher*. Affiliative behaviour is negatively related to brain IT concentration as indicated by the regression line in panel (*h*). For sample sizes, see table 1. For full statistical details, see table 3.

Affiliative behaviour was negatively related to brain IT levels (table 3 and figure 2*h*). There were marginally non-significant trends towards negative relationships between submission and brain AVT levels (table 3 and figure 2*e*), and restrained aggression and brain IT levels (table 3 and figure 2*d*). There were no relationships between the rates of other behavioural categories, and brain AVT or IT concentrations (table 3 and figure 2*a*–*c*,*f*,*g*).

## 5. Discussion

In this study, we measured biologically available AVT and IT in the brains of dominant and subordinate *N. pulcher* of both sexes. In contrast to previous work measuring AVT gene expression in *N. pulcher* [47] and in zebrafish [26], subordinate *N. pulcher* had higher actual concentrations of bioavailable AVT in their brains than did dominant fish. Measures of gene expression may not always correspond directly to measures of final bioactive nonapeptide concentrations, in part, because there are several steps in between mRNA production and the resultant final products. For example, proisotocin and provasotocin are proteolytically cleaved into the final active hormones IT and AVT, respectively. Furthermore, differences in gene expression reflect differences in nonapeptide production, whereas the level of available peptides may reflect differences in storage. For example, dominant and subordinate fish may differ in the degree to which AVT is being exported to the periphery versus being stored in the brain. In *O. mossambicus*, dominant individuals export a greater amount of AVT into their body where it stimulates the retention of urine, which is used as a social signal [34]. A similar effect could explain why we observed lower levels of AVT in the brains of dominant *N. pulcher*, despite the higher level of production suggested by the greater AVT gene expression reported earlier [47]. Our finding that AVT levels were higher in subordinate animals appeared to be driven primarily by the male subordinates having particularly high AVT concentrations in their brains (figure 1*a*), although, in general, brain AVT was not sex-dependent, as the level of AVT did not differ between male and female dominants. The results of Aubin-Horth *et al*. [47], in which dominant *N. pulcher* had higher expression of the AVT gene, were driven primarily by differences in the female dominant fish, so this is another way in which our results differ from previous findings using another technique. The fish used in this study came from the same study population and were housed in the same laboratory under similar conditions as the fish used in Aubin-Horth *et al*. [47], suggesting that differences in the origin or handling of the animals do not underlie the apparent differences in the results of these two studies.

The higher levels of AVT in the whole brains of subordinate *N. pulcher* compared with dominant fish suggest that AVT could be involved in the expression of submissive behaviour in this species. Both dominant and subordinate fish regularly produce aggressive behaviour, whereas submissive behaviour is rare in the dominants [46]. Therefore, the production of submissive displays is a key behavioural feature that distinguishes dominant from subordinate animals. AVT has been previously linked to the production of submissive behaviour in subordinates of another cichlid fish, the polygynous African cichlid, *A. burtoni*, where greater AVT mRNA expression in the parvocellular region of the preoptic area was found in subordinate males and the level of expression in this region correlated positively to the production of submissive behaviour [32]. Interestingly, we did not find a relationship between the level of AVT in the brains on *N. pulcher* and the rate at which the fish produced submissive displays. Furthermore, there was a statistical trend towards a negative rather than towards a positive relationship between AVT levels and submission. Collectively, these results suggest that a direct correspondence between AVT and submissive behaviour does not explain the status differences in AVT that we observed.

We did not find any association between the levels of biologically available IT and sex or social status in *N. pulcher*. Furthermore, there was no relationship between the amount of IT in the brain and the rate at which submissive or aggressive behaviours were produced, although there was a trend towards a negative relationship between brain IT level and the production of restrained aggressive displays. These results suggest that IT may not play a major role in the determination of social status in *N. pulcher*. Previous work on another cichlid, *O. mossambicus*, also did not find clear differences between the levels of IT in brains of dominants and subordinates [34]. To the best of our knowledge, this is the first measurement of brain IT in *N. pulcher* and adds to the small but growing literature on the behavioural role of IT in teleost fishes [18,19].

We found that the concentration of IT in the brain was correlated negatively with affiliative behaviour in *N. pulcher*. Affiliative behaviours involve approaching a social fellow, and interacting with that conspecific within close spatial proximity. These results are consistent with the hypothesis that IT is an important regulator of social approach in fishes [20,50]. Reddon *et al*. [50] found that exogenous IT delivered intraperitoneally reduced the tendency for *N. pulcher* to approach and affiliate with conspecifics, whereas an oxytocin antagonist increased this tendency. The results of these administration experiments fit with our findings in this study. The IT appears to have a negative effect on social approach and affiliation in *N. pulcher*. By contrast, Thompson & Walton [20] found that IT increased the tendency for goldfish to approach a conspecific. The difference in results could suggest species differences in the role of this peptide in regulating social approach, or may result from differences in methodology between the studies (intracerebroventricular infusions versus the measurement of endogenous levels). The cause of this discrepancy in results will require future work to resolve, ideally involving a combination of approaches on additional species. It is also worth considering that oxytocin has a known anxiolytic effect in mammals [54–57] although it is unknown if the same effect occurs in fishes. In many fish species, shoaling behaviour is an antipredator response [58], and shoaling may be evoked in stressful situations. If *N. pulcher* also cluster together with conspecifics in response to stress, then an anxiolytic effect of IT could, in principle, reduce the tendency to approach and affiliate with group members. This could partially account for our results and explain the reduced grouping tendency found in [50] following exogenous administration of IT. Further experimental study of the effects of IT on stress coping in *N. pulcher* in both social and non-social contexts will be essential to clarify this issue. Additional physiological measures, for instance, cortisol levels taken from the same animals would also be valuable.

We recorded all behaviour in terms of the number of discrete instances these behaviours were produced by each animal. Some of the behaviours (e.g. aggressive and submissive postures) that we observed vary in their duration and therefore might be better described by the total duration of the behaviour rather than by the frequency. In principle, gathering data on the duration of behaviours may offer higher-resolution behavioural data for future studies looking at the relationship between nonapeptide levels and behaviour in *N. pulcher* and other species. We also acknowledge that our study is limited to 9–11 individuals per group and that this relatively small sample size may account for the lack of significant relationships that we detected between brain nonapeptide levels and many of the behaviours we measured.

While the vast majority of studies of nonapeptide levels in the brains of fishes have used neuroanatomical or genomic proxies of nonapeptide production [26,31,32,47,59], we directly measured free biologically available nonapeptides in the brains of our animals. The discrepancy in the patterns observed between studies employing these different approaches emphasizes the complementary value of looking directly at biologically available free nonapeptides along with genomic and neuroanatomical techniques. The discrepancy suggests that this method should be included in further study of the role of nonapeptides in regulating behaviour [34]. One limitation of our current approach is that it involves averaging nonapeptide levels across the whole brain. The effects of the nonapeptides probably depend strongly both on their site of origin and the activated target region [60]. It is known in fishes that AVT cells in the parvocellular and magnocellular regions of the preoptic area show different correlations with behaviour [18,32]. Future studies that integrate multiple approaches within the same study system along with studies that provide brain-area-specific information about nonapeptide circuit function will be essential to fully elucidate the interplay between the production and circulating effects of the nonapeptide hormones and their role in regulating social behaviour.

In conclusion, dominant *N. pulcher* of both sexes have lower levels of biologically available AVT in their brains than do the subordinates. By contrast, we found no difference in the IT concentrations across dominance ranks. The individuals with higher concentrations of IT in their brains tended to be less affiliative than fish with lower levels of IT. Collectively, these results suggest that AVT may be an important regulator of social status in this species, and support the notion that IT may be involved in regulating social approach. Our results emphasize the value of directly measuring biologically available nonapeptide molecules in the brain.

## Supplementary Material

Supporting data

## Data accessibility

The dataset is available in the electronic supplementary material.
